# Winners and losers in the platform revolution

**DOI:** 10.1371/journal.pone.0340459

**Published:** 2026-02-10

**Authors:** J. Christopher Westland

**Affiliations:** University of Illinois Chicago, Chicago, Illinois, United States of America; SU: Salisbury University, UNITED STATES OF AMERICA

## Abstract

This study investigates the success and failure of open platform strategies in enhancing firm profitability and value, extending prior research by Parker and Van Alstyne (2018) and Parker et al. (2017). Utilizing an event study methodology, the research examines 52 open platform initiatives across various firms to assess their impact on firm value. The findings highlight a mixed outcome for open platform initiatives, where some events led to significant value creation, while others resulted in value destruction or no impact at all. Successful events often fostered an ecosystem of developers, accelerated innovation, and leveraged strategic advantages, while failures were attributed to strategic misalignment, perceived desperation, and inadequate open-source management. The study concludes that the value derived from open-sourcing depends heavily on aligning initiatives with core business strategies, offering clear value propositions, and engaging robust community management. These insights provide a nuanced understanding of how firms can effectively manage open platform strategies to balance innovation, control, and profitability.

## 1 Introduction

Open platform strategies represent one of the most significant business model innovations of the 21st century. Firms like Apple, Google, and Microsoft have created immense value by opening their technologies to external developers, fostering vast ecosystems of complementary innovation. Yet, for every success story, there are cautionary tales where platform initiatives have led to value destruction, strategic confusion, or have had no discernible market impact. This dichotomy presents a central puzzle: why do some open platform strategies succeed spectacularly while others fail? This study investigates this question by examining the financial market’s reaction to 52 open platform initiatives.

[1,2] studied information technology “platforms” noting that in the U.S. the majority of the largest public companies had adopted an “open platform” business model. They developed an elegant microeconomic model, arguing that open platform business models allowed firms to leverage external innovation to complement internal efforts [3,4]. They cited the firms in Table 1 as examples of successful open platform models. The success of platform models is often attributed to network effects, where the value of the platform increases for all users as more users and developers join. This can create a virtuous cycle of growth. However, the decision to open a platform is fraught with strategic trade-offs that can be understood through the lens of Transaction Cost Economics (TCE). While opening a platform lowers the firm’s internal development costs by leveraging external innovators, it simultaneously increases governance costs, the costs of managing, curating, and controlling the ecosystem to prevent opportunism and maintain quality [5]. This creates the fundamental tension between openness and control that lies at the heart of platform strategy.

**Table 1:** Companies mentioned in Parker and Van Alstyne 2018 (Specific dates of installation or significant changes were not provided for all platforms, but some key years for strategic changes were noted).

Company	Claimed open platform activity
Company	Claimed open platform activity
Apple	Mentioned in relation to its use of a closed technology platform model.
Cisco	Bundles network features developed by multiple developers.
Facebook	Opened to third
Google	Debuted as a public company in 2004 and has utilized an open platform business model.
Lockheed Martin	Aerospace
MakerBot	Absorbed developer innovations into its 3D platform
McCormick	Spices
Microsoft	Noted for absorbing developer innovations into its platform.
MySpace	Kept its development in house rather than adopting an open platform model.
Netscape	Utilized open platform strategies to spur innovation.
SAP	Uses an 18 to 24 month roadmap for developers before absorbing their innovations.
Threadless	T-shirts
Twitter	Built out a developer ecosystem before its initial public offering (IPO).
Uber	Funded to build a developer ecosystem in 2014.

Furthermore, the Resource-Based View (RBV) of the firm suggests that sustained competitive advantage comes from valuable, rare, and inimitable resources. An open platform can be such a resource, but only if managed correctly. If a firm opens its platform too much, it risks giving away the very intellectual property that forms its core advantage, allowing competitors to imitate its strategy and eroding its profitability (Eisenmann et al., 2011).

Therefore, the success or failure of an open platform initiative is not preordained but is a function of managerial decisions regarding the degree of openness, the governance structures in place, and the alignment of the platform strategy with the firm’s core business model. This research extends the foundational work in [1] by moving from microeconomic modeling to empirical testing. Utilizing an event study methodology, we assess how the market evaluates these strategic choices, providing a nuanced understanding of how firms can effectively manage open platform strategies to balance innovation, control, and profitability.

This research investigates the success and failure of open platform strategies in generating firm profitability and firm value. It extends research in [1,2] and several other papers and books which have studied information technology “platforms.” To study open platform strategies as a corporate value creator, the current research studied a set of 80 open platform introduction “events” (Appendix A) identified for firms highlighted in [1] (listed in Table 1) and assessed the contribution to firm wealth of each, using an event study methodology [6]. Event study methods require access to stock prices, so some of the firm events needed to be dropped. For example, MakerBot was taken private in 2013 and ceased to be a separate company in 2022; Twitter was taken private in 2022. These sorts of transitions reduced the number of events available for study to 52.

The research proceeds as follows. Sect 2 looks at prior research. Sect 3 sets up and conducts an aggregated event study, discussing the findings. Sect 4 segments individual event studies three ways: events that created wealth, events that destroyed wealth, and events that the market ignored. The 14 value creating events detailed in Appendix B; 15 value destroying events detailed in Appendix C; and 23 events that had no significant effect on value in Appendix D were analyzed for common factors that proved important in their success or failure. Sect 5 summarizes the findings for each of the claims made in [1], segmenting the findings for value-creating initiatives (winners) and value-destroying initiatives (losers). Sect 6 concludes with a summary and implications for management and strategy that can be drawn from these event studies.

## 2 Prior research

### 2.1 Platforms

Despite the prominence of open platforms, managing them brings unique challenges, as external developers respond differently than internal teams. [7] noted the lack of formal analysis on the ecosystem decisions of business platforms. The [1] microeconomic model suggested that firms can manage innovation by deciding how much intellectual property (IP) to give away, how to absorb developer IP, and how to condition these decisions on technical risks and IP reuse. These factors would then hopefully influence research and development (R&D) spillovers and future profits [8,9]. [2] noted that Apple, Facebook, and Microsoft had absorbed developer innovations into their platforms, ending the developers’ IP exclusivity to enhance the ecosystem’s functionality, sometimes to the detriment of community standards, user adoption and innovation spillovers [10,11].

Since the publication of [2], there has arisen an active research stream on digital platforms. [12] has suggested that cooperation between banks and fintech on digital platforms is already profoundly influencing the financial ecosystem, especially with digital asset clearing networks for cryptocurrency markets and a set of international markets that allow for trading and hedging with cryptocurrencies. [13] documents the manner in which platform enterprises use intangible assets to build monopoly and digital governance, profoundly changing the shape of society. [14] classifies technology-enabled multi-sided platforms in B2B relationships according to activities: transactional, innovation, and orthogonal. [15] discussed and explored proactive strategies and investments for platform launch and ecosystem growth. The exposition explores the dynamics of such investments similarly to the methods used in the current research, while identifying successfull strategies for initiation and growth of platforms. [5,16] reviewed cloudsourcing platforms for enhanced cost savings and service quality, finding they often fall short of promise. [17] investigated significant power asymmetries in digital platform ecosystems. [18] investigated sustainability in platforms, while [19] spoke on infrastructure. [20–22] review platformization in the Japanese context, with a focus on convenience store, and automotive platforms in Japan. In particular he challenges many of the fundamental tenets of platform capitalism, noting that rather than being digital artifacts, platforms have been a part of the society, culture in business of Japan for some time. Similarly, [23] reviewed evidence from Japan and the distinction between pipeline and platform advances, preferring the term accommodation for Japanese food platforms. [24] similarly seeks more precise definitions of platform capitalism, along with related terms, such as sharing economy, digital ecosystems, algorithmic decision making. [25] were concerned with eliciting how the platform impacts the exchange of value between supply chain members. [26] proposed and argued for a phased approach to digital transformation in manufacturing, because of the ambiguity of traditional metrics in properlly identifying and assessing the real drivers behind success on digital platforms. [25] leveraged prior research in information economics to assess how the platform impacts the exchange of value between supply chain members. [27] couched the ‘platform’ discussion in the larger sweep of technology evolution with a discussion of global dematerialization, AI, and the global stakeholder capitalism model of digital platforms, relying on arguments of evolutionary economics.

[2] further analyzed how much a platform should be opened to third-party developers and when to absorb their innovations, balancing between spurring growth and maintaining control. A platform that is too open might sacrifice direct profits and increase competition, while a too-closed platform might stifle innovation and limit user adoption [28,29]. [2] argue that optimal platform strategy involves fine-tuning these decisions based on production technology and code reusability, thereby maximizing both the firm’s and the public’s welfare. Overall, their research offered a comprehensive understanding of how to manage sequential innovation and platform openness [30,31]. These points are reiterated in a more discursive presentations in [2] as well as in articles that develop their ideas concerning platforms, e.g., [32–35]. Overall existing research offers a mixed portrait of open platform strategy – it can be profitable, but that is not guaranteed. Profitability requires luck and proper management. The [2] study makes specific claims in eight areas:

1. *Platform Openness vs. Control*: opening a platform can encourage innovation by allowing external developers to build on it. However, too much openness can reduce direct profits for the platform sponsor and increase competition. The model suggests a balance where enough openness stimulates third-party innovation without sacrificing too much control.
2. *Intellectual Property Duration*: the duration for which developers retain exclusive rights to their innovations is crucial. Longer durations can lead to higher royalties for both developers and the platform but may delay the conversion of innovations into a public good, hindering further innovation. The authors propose that optimal IP duration balances these factors, promoting ongoing innovation while ensuring that new ideas eventually become accessible to others.
3. *Sequential Innovation Model*: using a Cobb-Douglas production function, the [2] microeconomic model demonstrated “recursive innovation” (where each stage of innovation builds on the previous one) that affected the platform’s choices about openness and IP duration. Their model suggests that platforms need to offer enough value to developers to encourage participation but also consider the timing for absorbing these innovations into the platform.
4. *Welfare Optimization*: the [2] microeconomic model investigates welfare effects and argues that a social planner, in contrast to a profit maximizer, would aim for the greatest overall welfare, which may involve more openness and shorter IP durations than a profit-maximizing platform sponsor would choose.
5. *Technological Uncertainty*: when innovation success is less certain, platforms may opt for longer IP durations to mitigate risk and ensure returns on investment.
6. *Comparison with Open Standards*: the [2] microeconomic model compares platforms with open standards, where developers do not face restrictions. It highlights that while open standards can promote broad collaboration and innovation, they may lack the governance mechanisms to optimize spillovers and ensure coordinated development.
7. *Implications for Platform Strategy*: The findings provide insights for managers on designing contracts and governance structures that balance openness and control, leveraging external innovation while maintaining platform value.
8. *Policy and Regulatory Implications*: the [2] microeconomic model suggests that regulatory approaches should consider the dynamic nature of platform innovation and the benefits of managed ecosystems.

## 3 Event studies

Event studies have a long history, having over their history developed particular innovations that I rely on and which inform my construction of the set of event studies in this paper. Dolley [36] published a seminal event study on the price effects of stock splits. Subsequent authors [37–40] improved event study methodologies by removing general stock market price movements and separating out confounding events. Ball and Brown [41–43] introduced the methodology that is in use today. In the years since these pioneering event studies, a number of modifications have been developed relating to complications arising from violations of the statistical assumptions used in the early work and relate to adjustments in the design to accommodate more specific hypotheses [41,44–46].

Event studies appear in law and economics to measure the impact on the value of a firm of a change in the regulatory environment [47]. In legal liability cases, event studies have been used to assess damages [48,49]. In the majority of applications, the focus is on the effect of an event on the price of a particular class of securities of the firm, most often common equity.

MacKinlay [6] observed that the initial task of conducting an event study is to define the event of interest and identify the period over which market prices of the firms involved in this event will be examined, that is, the event window. This is typically set at around 10 days for financial studies, and this is the default window in the Wharton Research Data Services reference. Short event windows are suitable for stock market specific events, like earnings announcements. But in practice, the period of interest may need to be significantly expanded and periods prior to and after the event may also be of interest. After identifying the event, it is necessary to determine the selection criteria for the inclusion of a given firm in the study, the length of the time-series, and to discuss any potential biases that may have been introduced through the sample selection.

Warner [44] describes methodologies to improve the design and reliability of the studies over longer periods. Appraisal of the event’s impact requires a measure of abnormal return. The abnormal return is the actual ex post return of the asset over the event window minus the normal return of the asset over the event window. The normal return is defined as the expected return conditioned on the event. The constant mean return model assumes that the mean return of a given series of prices is constant through time. MacKinlay [6] notes that adding factors generally does not add a commensurate explanatory power and information to an event study, and thus recommends relatively simple models such as the CAPM model used in the current research.

### 3.1 Valuation models used in this study

Event studies assess the change in firm value “caused” by the event being studied. Typically, these involve a class of events, such as the open-sourcing of platforms studied here, on particular companies, at a resolution defined by the length of the event window.

Because open-sourcing of platforms is most useful to the technical staff and users of a particular platform, and because open-sourcing dates are not tightly controlled as would be, for example, earnings announcements, these open-sourcing events may take some time to percolate through the system. In the current study, we have assumed an event window ±90 days around the event date, which is considerably longer that might be encountered in event studies with financial announcements. The estimation window is also commensurately extended to one year. These values have been slightly modified in the individual event studies to accurately capture the immediacy of particular classes of events within this study.

Event studies assume that there is some “abnormal” return caused by each event, which of course requires a concept of “normal” return. This is assumed to be calculated by the Capital Asset Pricing Model (CAPM) [50,51]. The formulation of CAPM used in an event study is called the event study’s “market model.”

For firm *i* and event date *t* the abnormal return is:

$$AR_{i,t}=R_{i,t}-E(R_{i,t}|X_{t})$$ (1)

$E(R_{i,t}|X_{t})$ is calculated from the market model, a regression of the firm’s stock price time series {*X*_*t*_} against the corresponding market index {*M*_*t*_}, in our case the S&P500:

$$X_{i,t}={\hat{\alpha }}_{i}+{\hat{\beta }}_{i}M_{t}+\epsilon_{i,t}$$ (2)

for all t∈ estimation window

$$X_{i,\tau }={\hat{\alpha }}_{i}+{\hat{\beta }}_{i}M_{\tau }$$ (3)

for all τ∈ event window.

In the event window, the “abnormal” stock price ${\hat{X}}_{i,t}$ increase or decrease is:

$${\hat{X}}_{i,\tau }=X_{i,\tau }-{\hat{\alpha }}_{i}+{\hat{\beta }}_{i}M\tau$$ (4)

for all τ∈ event window

Abnormal returns $\left\{AR_{i,\tau }\right\}$ are computed from these “abnormal” differences. Event study statistics are computed for the cumulative abnormal return (CAR):

$$CAR_{i}=\sum_{\tau \in event.window}AR_{i,\tau }$$ (5)

As a general test of the value added by open-sourcing platforms, we can compute an aggregate CAR (CAAR)

$$CAAR=\frac{1}{N}\sum_{i=1}^{N}CAR_{i}$$ (6)

for *N* firms.

Event studies can adopt either the firm’s value-creation perspective or the investors’ “buy and hold” perspective. I have taken the value-creation perspective in this study. Given the generalist strategy focus of this research, I have taken a more relaxed threshold of 10% significance level for results. This will highlight potentially successful (or unsuccessful) open-sourcing events that can be further explored through discussion of the details of the open-sourcing, markets, potential value to the firm, and so forth.

The appropriate test for an event study depends on the behavior of the abnormal returns time series. In this study, all of the individual firm time-series exhibit significant heteroskedasticity, as well as cross-sectional correlation. The cross-sectional correlation is expected in stock prices, since overall market sentiment at any time, as well as macroeconomic influences, such as interest rates and money supply, will have a strong bearing on all stocks.

Where heteroskedasticity and cross-sectional correlation are high, as they universally are for the company stock price time-series used in this study, the Patell-z statistic is the preferred test statistic, where we test the hypothesis: *H*_0_:*E*(*CAAR*) = 0, i.e., the hypothesis that this class of events causes no change in firm value.

For my tests, I constructed an open-sourced platform event dataset of 80 events (listed in Appendix A, Table 3) acquired by perusing company websites and news releases on Google and other Internet news aggregators. I also constructed a stock price time-series dataset of 44,006 prices for the firms in Table 1 with prices acquired through the Wharton Research Data Services (WRDS) platform. The acquisition and use of this data complied with the terms and conditions of the WRDS service agreement. The price series ran from January 1, 1999 through June 30, 2024.

Prices were not collected where there was not a studied event within one year of the price data. Of the 80 events described in Appendix A, only 52 had sufficient stock market data for a complete event study. Some firms like MakerBot were not public, while others like Twitter, were only publicly traded for a portion of their existence. This subset of 52 platform open-sourcing events where stock market data was available served as the basis for the subsequent tests. Additionally, event announcements provided only approximate information on the release date; at other times, an open-source release might have happened over a week or more of time. Thus the event dates listed in Appendix A can be considered accurate only to within a month of resolution.

For any individual event, the financial impact of the event may be conflated with other, unobserved factors. Although this may hamper inference from a single event study, a collection of event studies from different times and different companies will not all be influenced by the same confounding factors. Thus I can reliably infer whether, in general, particular strategies and release conditions account for success or failure in open-source platform initiatives. These data-driven inferences then can provide reliable extensions and commentary to the insights provided in [1,2].

**Fig 1:**
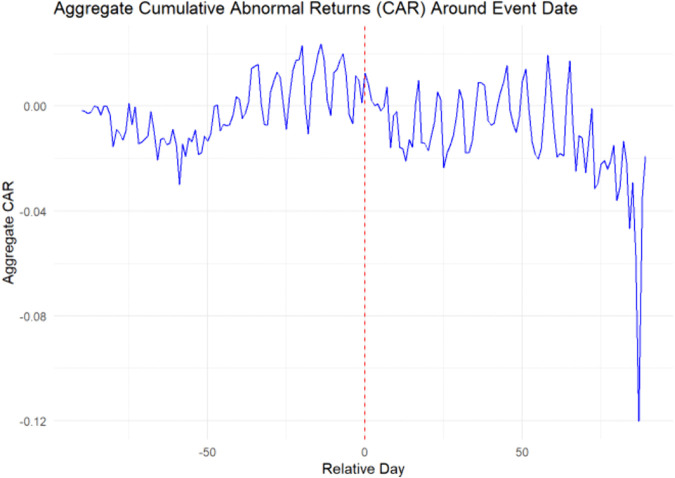
CAAR values in the event window.

The aggregate test yields the CAAR in the event window shown in the graph in Fig 1 where the Patell-z Statistic is −0.2534412 with a p-value of 0.7999273. The time-series graph in Fig 1 of the CAAR with event dates synchronized around the zero “Relative Day” shows a random fluctuation that leads us to conclude that there is no universal effect from adopting open-platforms. This is consistent with [2] admonition that management, luck and planning are also needed to insure a successful platform strategy. This is not unexpected given the complexities of managing open-sourcing, nor is it particularly enlightening.

Both [1,2] emphasized that the value of open-sourcing platforms arises only when their release is properly managed, and successful strategy requires a fine balance between giving away the firm’s hard earned assets, and attracting outside help in increasing the value of those assets. Thus we would expect a significant amount of variation in the success of individual open-sourcing of platforms, which requires analysis at the individual firm level. I performed individual platform event level tests performed in the next section.

## 4 Individual platform event-level tests

This section analyzes the value creation or destruction for each of the events in our 52 event subset, based on their Patell-z statistic and p-value. The Patell-z statistic is the preferred test statistic because of the high heteroskedctity and cross-sectional correlation in the price series for all of the stocks listed in Table 1.

Events were considered significant if their p-value≤0.10; this more relaxed critical value is due to the longer event window, which tended to allow more variation across the measured CAR. Value creating events had *Patell*–*z* > 0, i.e., CAR at the end of the event window was significantly greater than at the start; value destroying events had *Patell*–*z* < 0. I partitioned the results into three groups: (1) 14 value creating events detailed in Appendix B (Table 4); (2) 15 value destroying events detailed in Appendix C (Table 5); and (3) 23 events that had no significant effect on value in Appendix D (Table 6).

**Fig 2:**
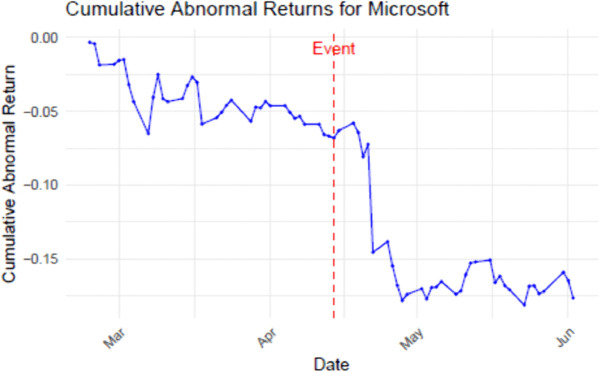
CAR values in the event window for Microsoft open-sourcing their visual Studio Code.

The Patell-z statistic measures the statistically significant change in CAR from beginning to end of the event window. As an illustration, Microsoft open-sourced their Visual Studio Code, a source code editor, on April 4, 2016. The graph of the CAR for this event appears in Fig 2. My event study returned a Patell-z statistic of −1.68 (i.e., it was negative, so reflects that value was destroyed) with a significant p-value of 0.092 (i.e., low chance that the event had no effect). The small Patell-z statistic suggests that perhaps because Microsoft’s stock price and capitalization are valuable, the relative loss was small. It also suggests that there was a delay before the event was generally recognized in the marketplace. The low time-resolution of the events being studied here suggests that the timing of announcements and effects are perhaps accurate to within a month, but probably not more accurate. This is the reason that I chose relatively large event and estimation windows for these individual platform event-level tests.

### 4.1 Common success themes

Appendix B lists the firms and their open-sourcing events that fell into the category of value-creating.

Across all these companies, the common threads in their open-sourcing strategies include fostering an ecosystem of developers, accelerating innovation, increasing transparency, gaining strategic advantages, and reducing costs. However, each company’s approach reflects its unique strategic priorities. Apple focuses on programming tools to attract developers to its platform; Google aims for broad adoption of its platforms and standards; Microsoft uses open source to support its cloud strategy, and Facebook builds tools to enhance performance and scalability. This diversity in approach highlights the different ways companies leverage open source as a strategic tool in the tech industry.

*Encouraging Ecosystem Growth:* Many companies, such as Apple and Google, open-sourced their platforms to encourage the development of an ecosystem around their products. For instance, when Apple open-sourced the Swift programming language in 2015, the goal was to increase adoption among developers and enhance the language through community contributions. Similarly, Google open-sourced Android and Chromium to foster a broad ecosystem of developers and manufacturers, contributing to the overall growth and success of these platforms. This move leverages the collective creativity and technical skills of external developers, leading to a more robust and feature-rich platform.

*Accelerating Innovation and Development:* Open-sourcing platforms often accelerates innovation by inviting contributions from a diverse set of developers worldwide. Apple’s open-sourcing of SwiftNIO, a cross-platform asynchronous event-driven network application framework, is another example. By making SwiftNIO open source, Apple aimed to accelerate innovation in networking frameworks across different platforms. This strategy benefits the company by bringing in fresh ideas, and code improvements, and identifying bugs faster than it could internally. Similarly, Google’s release of TensorFlow as an open-source platform brought advancements in machine learning frameworks by leveraging external contributions.

*Increasing Transparency and Trust:* By open-sourcing their platforms, companies also aim to build trust with developers and users. Transparency in software development, especially for platforms like Google’s Chromium or Apple’s FoundationDB, allows developers to understand how the software works, trust its security, and rely on its integrity. This is particularly significant in areas where privacy, security, and performance are critical. For example, when Google released Chromium, it was a move to assure users and developers of the transparency and security of their web browsing experience.

*Leveraging Open Source for Strategic Advantage:* Companies often use open source strategically to gain a competitive edge. For example, by open-sourcing TensorFlow, Google positioned itself as a leader in artificial intelligence and machine learning frameworks. This not only boosted Google’s credibility in the AI community but also ensured that a wide range of developers and companies adopted its framework, thereby indirectly supporting Google’s cloud and AI strategy. Similarly, Microsoft open-sourced .NET to counter competition from Java and other languages, aiming to make it a preferred choice for enterprise developers.

Reducing Development Costs: Open source can reduce development costs by shifting some of the burdens of innovation, testing, and debugging to the community. For instance, Apple open-sourced FoundationDB to leverage the community’s efforts in improving the distributed database, which can reduce internal costs associated with maintaining and updating the platform.

#### 4.1.1 Unique aspects.

##### 4.1.1.1 Apple:

- *Focus on Programming Languages and Frameworks:* Apple’s open-sourcing efforts have predominantly focused on programming languages (Swift) and development frameworks (SwiftNIO). This focus reflects Apple’s strategy to make its development tools and platforms more attractive to developers, encouraging them to build applications for Apple’s ecosystem (iOS, macOS). Swift, for example, was open-sourced to make it more accessible and attractive to a broader range of developers, not just those within Apple’s existing ecosystem.
- *Selective Open-Sourcing:* Apple has been relatively selective about what it open-sources. The open-sourcing of components like FoundationDB—a distributed database—was also strategic, aimed at improving the back-end services for its cloud infrastructure. Apple tends to open-source projects that have a direct benefit to its ecosystem and strategic goals.

##### 4.1.1.2 Google:

- *Broad Platform Strategy:* Google has been more aggressive and comprehensive in its open-source strategy, covering a range of platforms from mobile operating systems (Android) to web browsers (Chromium) and machine learning frameworks (TensorFlow). Google’s approach reflects its strategy of creating platforms that are widely adopted and becoming standards in their respective fields.
- *Community and Collaboration Focus:* Google’s approach to open source also heavily emphasizes community and collaboration. This is evident from its Android strategy, where it built an ecosystem involving multiple manufacturers, developers, and service providers. By making Android open-source, Google positioned itself as a leader in mobile operating systems while ensuring widespread adoption and minimizing development costs.

##### 4.1.1.3 Microsoft:

- *Transformation through Open Source:* Microsoft’s recent open-source efforts, such as open-sourcing .NET, reflect a significant cultural shift from its historical stance against open source. This transformation aligns with Microsoft’s broader strategy to be seen as a more open and collaborative company, especially under the leadership of Satya Nadella. Open-sourcing .NET was intended to expand its use among developers and encourage development on the Azure cloud platform. Open Source as a Cloud Strategy: For Microsoft, open source is a key part of its cloud strategy. By open-sourcing key frameworks and tools, Microsoft encourages developers to use its platforms and services, indirectly promoting its Azure cloud offerings.

##### 4.1.1.4 Facebook:

- *Building Developer Tools and Frameworks:* Facebook’s open-source efforts have largely focused on developer tools and frameworks like React and GraphQL. These projects are designed to enhance web and mobile development, reflecting Facebook’s roots in web development and its strategy to foster a community of developers who are invested in its ecosystem.
- *Focus on Performance and Scalability:* Facebook’s open-source contributions also focus on performance and scalability, critical aspects for handling the massive data and user interaction volumes Facebook deals with. Projects like GraphQL, which allows for more efficient data fetching, are part of this strategy.

### 4.2 Reasons for failure

Appendix C lists the firms and their open-sourcing events that fell into the category of value-destroying.

The destruction of wealth and stock value following these open-sourcing events can be attributed to several common factors: strategic misalignment, perceived signs of desperation or weakness, underestimation of the complexities involved in open-source management, loss of competitive advantage, and negative market perception of strategic shifts. Each company’s unique approach and historical context played a role in how these actions were perceived by the market. Ultimately, for open-sourcing initiatives to be successful, they must align closely with the company’s core strategy, offer clear value propositions to both the company and the community, and be supported by robust community engagement and management efforts.

#### 4.2.1 Misalignment with core business strategy.

A frequent issue across these events is a misalignment between the open-sourcing decision and the company’s core business strategy. For example, Apple’s decision to open-source the HomeKit Accessory Development Kit (ADK) to encourage wider adoption did not align well with its traditional business model, which is centered around maintaining control over its ecosystem. This move likely created confusion about Apple’s strategic direction, undermining investor confidence and leading to a decline in stock value. When open-sourcing efforts do not clearly support or enhance a company’s core business, the market often reacts negatively due to perceived strategic missteps.

#### 4.2.2 Perceived weakness or desperation.

Another theme is that the market often perceives open-sourcing a platform as a sign of weakness or desperation, especially when the open-sourced technology is not thriving or is considered obsolete. For instance, Microsoft’s open-sourcing of .NET Core and TypeScript, although eventually successful in some developer circles, was initially seen as an attempt to regain relevance in a market dominated by more modern and flexible languages and frameworks. The perception that a company is giving away its technology due to its inability to compete effectively can lead to a loss of investor confidence and a subsequent drop in stock prices.

#### 4.2.3 Underestimation of open source costs and community management.

Companies may underestimate the costs and complexities involved in managing an open-source project. Microsoft’s initial forays into open-sourcing, such as with .NET Core, required significant investment in community management, documentation, and support. Failure to properly allocate resources or manage these aspects can lead to poor adoption and negative sentiment from the developer community, further impacting stock value. When companies do not fully commit to the open-source ethos or fail to engage adequately with the community, the perceived benefits of open-sourcing are diminished, and market reactions tend to be negative. Loss of Competitive Advantage and IP Concerns:

Open-sourcing can also lead to the loss of competitive advantage, especially if the technology being open-sourced was a key differentiator for the company. By making critical technology available to everyone, companies can erode their unique market position. Additionally, there are risks associated with intellectual property (IP) exposure, including the potential for competitors to capitalize on the open-sourced technology without bearing the initial development costs. For example, Apple’s open-sourcing of certain security frameworks raised concerns about the potential misuse of its technology and the loss of its competitive edge in secure environments. Negative Market Perception of Strategic Shifts:

Strategic shifts, particularly those perceived as sudden or reactionary, often unsettle investors. For instance, Apple’s decision to collaborate with Google to enhance TensorFlow Lite for iOS might have been seen as an acknowledgment of Google’s superiority in AI and machine learning, which could undermine Apple’s reputation for innovation and technological leadership. Such moves can create uncertainty about a company’s strategic direction and lead to negative market reactions.

#### 4.2.4 Unique aspects of each company’s approach.

##### 4.2.4.1 Apple:

*Control vs. Openness:* Apple’s brand and strategy have long been associated with a closed, controlled ecosystem. Moves to open-source certain components, such as the HomeKit ADK or the Password Manager Resources, were seen as out of character and potentially indicative of strategic confusion. Investors likely viewed these actions as diluting Apple’s tightly controlled ecosystem, leading to concerns over its future strategic direction and market differentiation. Security and Privacy Concerns: Apple’s open-sourcing efforts, especially those related to security (like the Password Manager Resources), might have raised concerns about the potential for security breaches or misappropriation of sensitive technologies. The market often views security and privacy as integral to Apple’s value proposition, and any move perceived as weakening this aspect could negatively impact stock value.

##### 4.2.4.2 Microsoft:

*Pivot to Open Source:* Microsoft’s decision to open-source technologies such as .NET Core and TypeScript was initially seen as a radical shift from its historically proprietary stance. This pivot was viewed with skepticism by investors, who may have perceived it as a sign of Microsoft’s waning influence in the face of growing competition from more open, developer-friendly ecosystems like those of Google or open-source communities. Market Misalignment: Initially, Microsoft’s open-sourcing efforts were not clearly aligned with its revenue model, which heavily relied on software licensing. The shift to open-source, without a clear monetization strategy tied to cloud services or other revenue-generating areas, led to concerns about Microsoft’s ability to sustain its financial performance, causing negative reactions in the stock market.

##### 4.2.4.3 Other Potential Sources of Failure in Open-source Platform Initiatives:

*Community Engagement Failures:* Other companies might have failed to engage with the open-source community effectively, leading to poor adoption of their open-sourced platforms. This lack of engagement could manifest in inadequate documentation, slow response times to community contributions, or a lack of clear leadership and vision for the open-sourced project. Such failures can lead to a lack of trust and support from the developer community, which can be detrimental to the platform’s success and the company’s stock value. Case Studies of Wealth Destruction Apple and the Open-Sourcing of HomeKit ADK:

Apple’s open-sourcing of the HomeKit ADK was intended to accelerate the adoption of its home automation framework. However, the market perceived this as a lack of commitment to its ecosystem’s exclusivity and a potential dilution of its brand’s perceived value. The subsequent stock value decline highlights investor concerns about strategic coherence and competitive positioning. Microsoft and Open-Sourcing of .NET Core:

The open-sourcing of .NET Core was part of Microsoft’s broader strategy to engage with the open-source community and promote cross-platform development. However, the initial market reaction was negative, as investors perceived it as an acknowledgment of the company’s declining dominance in the software market and a potentially costly shift away from its lucrative licensing model.

### 4.3 Reasons events had no impact

Appendix D lists the firms and their open-sourcing events that seem neither to have created value, nor destroyed it. In this section I discuss why open-sourcing their platforms, which must have seemed strategically important to managers, seemed not to have impacted the value of these firms.

#### 4.3.1 Lack of strategic importance.

A common factor across these cases is that the platforms open-sourced were not deemed strategically critical to the core business of the companies involved. For instance, when Apple open-sourced WebKit or ResearchKit, these moves did not pertain to Apple’s core revenue-generating products such as iPhones or Macs. The market likely perceived these as peripheral initiatives that did not significantly alter Apple’s business model or competitive positioning. The strategic relevance of a platform significantly affects how the market reacts; if a platform is not central to the company’s future growth or operations, its open-sourcing is less likely to impact stock prices.

The market’s indifference to certain open-sourcing events can be attributed to a combination of factors, including the lack of immediate financial impact, alignment with existing strategies, low visibility, and the perception of these initiatives as non-transformative. While these open-sourcing efforts may still provide long-term strategic benefits, such as fostering developer ecosystems or reinforcing market positions, their immediate impact on firm valuation and wealth was limited, resulting in little to no effect on stock prices.

#### 4.3.2 Mature or saturated markets.

Another theme is the maturity or saturation of the markets involved. For example, by the time Google open-sourced TensorFlow, the market for machine learning frameworks was already populated with strong competitors, including open-source alternatives like Theano and Torch. The market’s saturation meant that Google’s move, while significant within the developer and academic communities, did not translate into a broader impact on market valuation. In mature markets, incremental innovations or changes, such as open-sourcing an already existing tool, are less likely to create significant new economic value. Expectation of Open Source:

In several cases, the market may have already anticipated the move towards open-sourcing. For companies like Google and Facebook, which have a strong track record of contributing to open source, additional contributions may not surprise investors or analysts. For instance, the open-sourcing of TensorFlow might have been viewed as a natural progression in Google’s strategy to dominate the machine learning space and support its cloud services. Similarly, Apple’s decision to open-source frameworks like ResearchKit and CareKit could have been seen as expected moves to foster innovation and collaboration in health tech, a growing but not revolutionary field for the company. Lack of Immediate Revenue Impact:

Many of the platforms open-sourced did not directly contribute to immediate revenue streams. For instance, Apple’s WebKit or Google’s TensorFlow are not direct revenue generators; rather, they serve as enablers for broader ecosystems. Open-sourcing such platforms does not change the immediate financial outlook or cash flows of the companies, leading to a muted market response. Investors are more likely to respond to initiatives that have clear and immediate implications for revenue growth or profitability, rather than long-term ecosystem or strategic plays. Technical or Niche Market Focus:

Many open-sourced platforms addressed specific technical needs or niche markets rather than broad consumer or enterprise markets. For example, Apple’s Darwin source code release was primarily of interest to developers and did not have a significant impact on the broader market. Similarly, TensorFlow, while important for AI researchers and developers, did not have immediate implications for the broader consumer market that might drive significant stock price changes. When the open-sourced platform caters to a niche market, the financial impact on the company as a whole is often limited, leading to market indifference.

#### 4.3.3 Unique aspects of each company’s approach.

##### 4.3.3.1 Apple:

*Selective Open-Sourcing:* Apple’s open-sourcing efforts, such as those for WebKit, ResearchKit, and CareKit, have generally been selective and targeted at specific communities. These efforts are designed to enhance platform usability and attract developers without directly impacting Apple’s hardware or core software revenue streams. This selective approach has led to a perception that these initiatives are more about ecosystem development than revenue generation, which may explain the market’s indifference.

*Focus on Enhancing Developer Ecosystem:* Apple’s open-sourcing of WebKit, the browser engine behind Safari, was primarily aimed at developers and did not carry immediate financial implications. Similarly, ResearchKit and CareKit were aimed at fostering innovation in health-related apps but were not seen as revenue drivers. The market likely viewed these initiatives as beneficial to the developer community but not directly impactful to Apple’s bottom line. Google:

*Reinforcing Existing Strengths:* Google’s open-sourcing of TensorFlow reinforced its position in machine learning and AI, a field where it was already a leader. The market may have perceived this move as a continuation of Google’s established strategy rather than a new, impactful initiative. TensorFlow’s open-source release was significant in the tech community but did not fundamentally alter Google’s revenue model or competitive landscape, hence the muted market response.

*Incremental Rather than Transformative:* For Google, many of these open-source initiatives were seen as incremental improvements or natural extensions of existing strategies rather than transformative changes that would drive new revenue streams or significant cost savings. As a result, the financial markets may not have seen these moves as game-changers, leading to indifference in stock price movement.

##### 4.3.3.2 Microsoft:

*Supporting Broader Cloud Strategy*: Microsoft’s open-sourcing moves, such as those involving .NET, were largely seen as supporting its broader cloud strategy, particularly Azure. While these initiatives were strategically sound, they were also seen as supporting existing revenue streams rather than creating new ones. The market’s lack of reaction can be attributed to this perception of continuity rather than change.

##### 4.3.3.3 Facebook:

*Developer-Centric Releases:* Facebook’s open-source initiatives often focus on developer tools and frameworks, such as React or GraphQL. These tools, while important for developer adoption and community building, are not direct revenue generators and have limited impact on Facebook’s core advertising business. As a result, these releases are often seen as tactical rather than strategic, resulting in minimal market impact. Reasons for Market Indifference No Immediate Financial Impact:

The open-sourced platforms in these cases did not directly affect the companies’ immediate financial performance. Without a clear link to revenue or cost savings, the market tends to disregard such initiatives as they do not alter the company’s short-term financial outlook. Perceived as Expected Moves:

Many open-sourcing decisions were perceived as expected or logical extensions of the companies’ strategies. When such moves are anticipated by the market, they do not create a surprise factor that might drive significant stock price changes. Low Visibility and Awareness:

Some open-sourcing initiatives might have had low visibility outside of niche communities. For instance, while TensorFlow is widely recognized in AI circles, it might not have the same level of recognition or perceived impact among the broader investor community, contributing to the lack of significant market reaction. Unclear Strategic Benefits:

In cases where the strategic benefits of open-sourcing were unclear or indirect, the market response was likely muted. Investors may have struggled to understand how these initiatives fit into the broader strategic goals of the company, leading to uncertainty and indifference.

## 5 Conclusions

In this and the next section I explore the eight claims presented by [1] in their analysis of platform strategies and innovation dynamics, first for the successful initiatives, and then for those that failed. These claims are examined against the findings of my event study that assessed the impact of open-platform initiatives by the firms cited in [1] and listed in Table 1. Here is the support or lack thereof provided by my successful (Appendix B) or value-destroying (Appendix C) event study for each open-platform claim made in [1].

### 5.1 Successful (value-creating) open-platform initiatives

*1. Platform Openness vs. Control*

*Claim:* Opening a platform can encourage innovation by allowing external developers to build on it. However, too much openness can reduce direct profits for the platform sponsor and increase competition.

*Evaluation:* Supported. The event study confirms that firms like Apple, Google, and Microsoft strategically open-sourced certain platforms to foster an ecosystem of developers. This encouraged innovation and reduced costs by leveraging external contributions. However, these companies maintained control over strategic components to prevent excessive competition and protect profits. For instance, Apple’s selective open-sourcing aligns with maintaining a balance between openness and control, as the claim suggests.

*2. Intellectual Property Duration*

*Claim:* The duration for which developers retain exclusive rights to their innovations is crucial. Longer durations can lead to higher royalties but may delay the conversion of innovations into a public good, hindering further innovation.

*Evaluation:* Partially Supported. While the event study does not directly address IP duration, the strategies observed suggest that firms favor quicker public access to drive innovation rather than longer exclusive rights. Companies like Google and Microsoft have rapidly open-sourced key technologies (e.g., TensorFlow, .NET) to accelerate innovation and adoption, which indicates a preference for shorter IP durations to stimulate ongoing innovation rather than maintaining exclusivity for extended periods.

*3. Sequential Innovation Model*

*Claim:* Platforms need to offer enough value to developers to encourage participation while also considering the timing for absorbing these innovations into the platform.

*Evaluation:* Supported. The event study supports this claim by showing how companies like Apple and Google structured their open-source initiatives to ensure they remained attractive to developers, fostering a continuous cycle of innovation. Google’s strategy of open-sourcing Android and Chromium illustrates a model of sequential innovation where each stage builds on previous contributions, aligning with [1]’s concept of recursive innovation.

*4. Welfare Optimization*

*Claim:* A social planner, in contrast to a profit maximizer, would aim for the greatest overall welfare, potentially involving more openness and shorter IP durations.

*Evaluation:* Not Directly Supported. The event study focuses on corporate strategies that increase firm value rather than welfare optimization from a social planner’s perspective. While firms like Google and Microsoft have employed open-source strategies that indirectly benefit broader developer communities, their primary objective remains maximizing their strategic advantage and profits, not necessarily optimizing overall welfare as a social planner would.

*5. Technological Uncertainty*

*Claim:* When innovation success is less certain, platforms may opt for longer IP durations to mitigate risk and ensure returns on investment.

*Evaluation:* Not Supported. The event study suggests that companies facing technological uncertainty, such as in nascent AI and machine learning fields, prefer to open-source their platforms (e.g., Google’s TensorFlow) rather than extend IP durations. This approach appears to mitigate risk by fostering community collaboration and rapid iteration, contrasting with the claim’s suggestion that longer IP durations are preferred under uncertainty.

*6. Comparison with Open Standards*

*Claim:* While open standards can promote broad collaboration and innovation, they may lack the governance mechanisms to optimize spillovers and ensure coordinated development.

*Evaluation:* Supported. The event study findings resonate with this claim, particularly regarding the governance role of companies like Apple, Google, and Microsoft in managing their open-source projects. These companies often maintain significant control over project directions, demonstrating a need for governance mechanisms to optimize outcomes, in contrast to fully open standards.

*7. Implications for Platform Strategy*

*Claim:* Insights for managers on designing contracts and governance structures that balance openness and control, leveraging external innovation while maintaining platform value.

*Evaluation:* Supported. The event study clearly shows that firms use open-sourcing strategically to enhance platform value while maintaining control. Apple’s selective open-sourcing and Google’s comprehensive approach illustrate how different governance structures can be designed to balance openness with strategic control, aligning well with this claim.

*8. Policy and Regulatory Implications*

*Claim:* Regulatory approaches should consider the dynamic nature of platform innovation and the benefits of managed ecosystems.

*Evaluation:* Partially Supported. While the event study does not delve into regulatory implications, it does suggest that companies’ strategic use of open-source initiatives aligns with a dynamic approach to innovation. This supports the idea that regulators should consider the fluid and evolving nature of platform ecosystems when designing policies, as platforms can provide public benefits while maintaining control over strategic elements.

### 5.2 Failed (value-destroying) open-platform initiatives

*1. Platform Openness vs. Control*

*Claim:* Opening a platform can encourage innovation by allowing external developers to build on it. However, too much openness can reduce direct profits for the platform sponsor and increase competition.

*Evaluation:* Supported. The event study findings suggest that excessive openness can indeed undermine a firm’s strategic position and lead to value destruction. For instance, Apple’s decision to open-source the HomeKit Accessory Development Kit (ADK) did not align well with its historically closed ecosystem approach, leading to confusion about its strategic direction and resulting in a negative market reaction. This outcome aligns with [1]’s claim that a balance between openness and control is crucial to avoid reduced profits and increased competition.

*2. Intellectual Property Duration*

*Claim:* The duration for which developers retain exclusive rights to their innovations is crucial. Longer durations can lead to higher royalties but may delay the conversion of innovations into a public good, hindering further innovation.

*Evaluation:* Not Directly Supported. The event study did not specifically address the issue of IP duration. However, it highlighted that companies failing to align open-source initiatives with their core strategies faced negative market reactions. The lack of a clear strategy or benefit to the company in the long term might reflect concerns similar to those about IP duration—where longer control does not necessarily equate to better outcomes, especially if it leads to misalignment with business objectives.

*3. Sequential Innovation Model*

*Claim:* Platforms need to offer enough value to developers to encourage participation while considering the timing for absorbing these innovations into the platform.

*Evaluation:* Partially Supported. The event study findings suggest that misalignment in strategic timing can lead to negative outcomes. For example, Microsoft’s open-sourcing of .NET Core was initially perceived as a desperate attempt to regain relevance. This misalignment in timing and perceived value to developers initially led to a loss of investor confidence, supporting the need for careful timing in absorbing innovations as [1] suggests.

*4. Welfare Optimization*

*Claim:* A social planner, in contrast to a profit maximizer, would aim for the greatest overall welfare, potentially involving more openness and shorter IP durations.

*Evaluation:* Not Supported. The firms in the event study primarily acted as profit maximizers, and their open-sourcing decisions were evaluated on the basis of their impact on firm value rather than overall welfare. The negative reactions to open-sourcing that seemed misaligned with strategic objectives suggest that these companies were not optimizing for broader social welfare but rather for direct competitive and financial advantages. Thus, the claim that firms would adopt more open strategies under a welfare optimization model is not supported by the findings.

*5. Technological Uncertainty*

*Claim:* When innovation success is less certain, platforms may opt for longer IP durations to mitigate risk and ensure returns on investment.

*Evaluation:* Not Supported. The event study shows that companies facing uncertainty often made strategic errors by choosing to open-source without fully understanding the market dynamics or the potential negative perception of such moves. For instance, when Microsoft open-sourced .NET Core, it was initially seen as an attempt to catch up rather than a strategic strength, leading to a perception of uncertainty and resulting in a negative market response. This contrasts with the claim that longer IP durations would mitigate risk; rather, openness without clear alignment and management led to increased perceived risk.

*6. Comparison with Open Standards*

*Claim:* Open standards can promote broad collaboration and innovation but may lack governance mechanisms to optimize spillovers and ensure coordinated development.

*Evaluation:* Supported. The negative outcomes of certain open-sourcing events in the study suggest that without strong governance, open standards can fail to deliver the expected value. Microsoft’s initial struggles with community management for .NET Core highlight the importance of governance in managing open-source projects, as poor management led to negative sentiment and poor adoption. This aligns with the claim that open standards, while collaborative, require strong governance to maximize benefits.

*7. Implications for Platform Strategy*

*Claim:* Insights for managers on designing contracts and governance structures that balance openness and control, leveraging external innovation while maintaining platform value.

*Evaluation:* Supported. The event study findings provide strong support for this claim. Companies that failed to balance openness with control, like Apple with its HomeKit ADK, experienced negative market reactions. This underscores the importance of designing strategic governance structures that manage open-source initiatives effectively, ensuring they align with overall platform value and strategic goals.

*8. Policy and Regulatory Implications*

*Claim:* Regulatory approaches should consider the dynamic nature of platform innovation and the benefits of managed ecosystems.

*Evaluation:* Partially Supported. While the event study did not directly address regulatory implications, it highlighted the complexities involved in open-sourcing strategies and the potential for negative outcomes when these are poorly managed or misaligned with core business objectives. This suggests that regulatory bodies should indeed consider the dynamic and strategic nature of platform innovation when designing policies, as poorly conceived open-sourcing can destroy value rather than create it.

### 5.3 Discussion

I explored the eight claims presented by [1] in their analysis of platform strategies and innovation dynamics, first for the successful initiatives, and then for those that failed. These claims were examined against the findings of my event study that assessed the impact of open-platform initiatives by the firms cited in [1] and listed in Table 1. Table 2 summarizes the findings from my event study analyses on whether or not individual open-platform initiatives created or destroyed firm wealth.

**Table 2:** Summary of event study findings.

Claim	Successes (Appendix B)	Failures (Appendix C)
Platform Openness vs. Control	Supported	Supported
Intellectual Property Duration	Supported	Not Directly Supported
Sequential Innovation Model	Supported	Partially Supported
Welfare Optimization	Not Directly Supported	Not Supported
Technological Uncertainty	Not Supported	Not Supported
Comparison with Open Standards	Supported	Supported
Implications for Platform Strategy	Supported	Supported
Policy and Regulatory Implications	Partially Supported	Partially Supported

These claims in [1] were examined against the findings of my event study that assessed the impact of open-platform initiatives by the firms cited in Table 1. Overall, the event study findings support for most of the claims made by [1], particularly those related to balancing openness and control, fostering ecosystems, leveraging external innovation, and the need for governance mechanisms in open standards. However, the claims regarding welfare optimization and preferences for longer IP durations under uncertainty are less supported by the study’s conclusions, which focus on corporate strategies to maximize firm value and foster rapid innovation through openness.

Certain of my event studies identified cases where open-sourcing led to value destruction, often due to misalignment with core business strategies, perceived signs of desperation, and mismanagement of open-source projects. Here, we evaluate whether these claims are supported by the event study findings, focusing on instances where open-platform initiatives did not create value and, in some cases, even led to a decline in stock value. On the other hand, wealth was created by open-source platform initiatives when those related to the need for strategic alignment, balanced openness, and strong governance in open-source initiatives. However, some claims, such as those related to welfare optimization and IP duration preferences under uncertainty, are not directly supported by the event study outcomes. My analyses emphasizes that open-sourcing must be carefully managed and aligned with core business strategies to avoid value destruction and maximize potential benefits.

### 5.4 Contribution to the literature

This study makes several key contributions to the literature on platform strategy and innovation. First, while the foundational work of Parker & Van Alstyne (2018) and others provides elegant microeconomic models of platform dynamics, this research provides one of the first large-scale empirical tests of their central claims. By employing an event study methodology across 52 distinct platform initiatives, we move from theoretical proposition to market-based evidence, showing which strategies create or destroy firm value in practice.

Second, these findings refine existing theory. We provide strong support for the theoretical claim that a careful balance between platform openness and control is critical for value creation. However, our results challenge the proposition that firms facing technological uncertainty should prefer longer IP durations; instead, we find that in nascent fields like AI, firms often open-source key technologies to foster community collaboration and mitigate risk through rapid iteration. This adds an important nuance to our understanding of platform strategy under uncertainty.

Finally, by segmenting events into “winners,” “losers,” and those with no impact, this study provides a granular view that aggregated studies often miss. This demonstrates that the “open platform” label is too coarse; the strategic intent, market context, and execution of the initiative are what ultimately determine its outcome. This provides a clear guide for future research to move beyond whether firms should adopt open platforms, and toward how they should be designed and managed for success.

## Supporting information

Appendix A Open-Platform “Events” for Companies in Table 1. (PDF)

Appendix B Value-creating events. (PDF)

Appendix C Value-destroying events. (PDF)

Appendix D Events that did not impact firm value. (PDF)
