# Supplementary material for: Winners and losers in the platform revolution
Source: PLoS One. 2026 Feb 10;21(2):e0340459. doi: 10.1371/journal.pone.0340459 (PMC12890110; doi:10.1371/journal.pone.0340459)
Supplement: Appendix A — (PDF) [file pone.0340459.s001.pdf]

Appendix A: Open-Platform “Events” for Companies in Table 1

Table 1: Open Platform "Events"

Company	Date	Event
Apple	1999-01-01	Apple first released the source code for Darwin, the Unix-based core of macOS.
Apple	2003-06-13	Apple open-sourced WebKit, the browser engine used in Safari.
Google	2007-11-05	Google announced the open-sourcing of the Android OS.
Google	2008-07-07	Google released Protocol Buffers as an open-source project.
Google	2008-09-02	Google released Chromium, the open-source project behind the Chrome browser.
Twitter	2010-06-01	Twitter began contributing to Apache Mesos, a cluster manager that provides efficient resource isolation and sharing.
Google	2010-10-20	Google open-sourced AngularJS, a web application framework.
Twitter	2011-07-18	Twitter open-sourced Finagle, an extensible RPC system for the JVM used for building asynchronous, distributed systems.
Twitter	2011-08-19	Twitter open-sourced Bootstrap, a popular front-end framework for web development.
Cisco	2012-01-01	Cisco began contributing to the OpenStack project, a cloud computing platform.
Google	2012-03-10	Google open-sourced the Go (Golang) programming language.
Twitter	2012-04-19	Twitter open-sourced Scalding, a Scala API for Cascading, which is a framework for building robust data processing workflows on Hadoop.
Cisco	2012-06-04	Cisco launched the Open Network Environment (ONE) to encourage open standards and programmability in networking.
Twitter	2012-07-12	Twitter made significant contributions to the Hadoop ecosystem, particularly around scalability and integration.
Twitter	2012-08-14	Twitter open-sourced Zipkin, a distributed tracing system for service-oriented architectures.
Twitter	2012-08-21	Twitter open-sourced Bower, a package manager for the web.
Microsoft	2012-10-01	Microsoft open-sourced TypeScript, a typed superset of JavaScript.
Cisco	2013-04-08	Cisco was a founding member and major contributor to the OpenDaylight project, an open-source SDN (Software-Defined Networking) controller.
Facebook	2013-04-24	Facebook open-sourced Buck, a build system for Android.
Facebook	2013-05-29	Facebook open-sourced React, a JavaScript library for building user interfaces.
Twitter	2013-06-15	Twitter open-sourced Twemproxy, a fast and lightweight proxy for Memcached and Redis protocols.
Facebook	2013-11-06	Facebook open-sourced PrestoDB, a distributed SQL query engine for big data.
Facebook	2013-11-22	Facebook open-sourced RocksDB, a high-performance key-value store.
SAP	2013-12-12	SAP open-sourced OpenUI5, a JavaScript UI framework for building responsive web applications.
SAP	2014-01-01	SAP began contributing to the Cloud Foundry Foundation as a platinum member and later open-sourced various components related to SAP Cloud Platform.

Google	2014-06-07	Google open-sourced Kubernetes, a container orchestration platform.
Microsoft	2014-11-12	Microsoft open-sourced .NET Core, a cross-platform framework for building applications.
Facebook	2015-02-02	Facebook open-sourced Fresco, an image loading library for Android.
Apple	2015-03-09	Apple announced and released ResearchKit as an open-source framework for medical research.
Google	2015-03-19	Google open-sourced Bazel, a build and test tool.
Facebook	2015-03-26	Facebook open-sourced React Native, a framework for building native apps using React.
Facebook	2015-06-10	Facebook open-sourced Infer, a static analysis tool for finding bugs in Java and C code.
Facebook	2015-07-07	Facebook released GraphQL, a data query language, as an open-source project.
Google	2015-11-09	Google open-sourced TensorFlow, its machine learning framework.
Apple	2015-12-03	Apple open-sourced the Swift programming language.
Cisco	2015-12-17	Cisco joined the Hyperledger project to support blockchain technology and contributed to its development.
Microsoft	2016-01-13	Microsoft open-sourced ChakraCore, the core of the Chakra JavaScript engine.
Cisco	2016-03-09	Cisco joined Microsoft in supporting SONiC, an open-source network operating system.
SAP	2016-03-24	SAP contributed the Dirigible project to the Eclipse Foundation, an open-source application development platform.
Microsoft	2016-04-14	Microsoft open-sourced Visual Studio Code, a source code editor.
Apple	2016-04-21	Apple released CareKit, an open-source framework for healthcare apps.
Twitter	2016-04-27	Twitter open-sourced Cuckoo Filter, a high-performance, probabilistic data structure for fast approximate set-membership queries.
Twitter	2016-06-15	Twitter open-sourced Heron, a real-time analytics platform that is the successor to Apache Storm.
Google	2016-08-15	Google released the source code for its experimental operating system, Fuchsia.
Microsoft	2016-08-18	Microsoft open-sourced PowerShell, a cross-platform task automation framework.
Google	2017-01-10	Google contributed the Dataflow SDK to the Apache Software Foundation, forming Apache Beam.
Facebook	2017-01-19	Facebook open-sourced PyTorch, a deep learning framework.
SAP	2017-02-21	SAP open-sourced Project "Piper," a continuous integration and delivery (CI/CD) tool for DevOps.
Cisco	2017-03-15	Cisco contributed to the Open Security Controller project under the Linux Foundation to provide security orchestration.
Uber	2017-04-20	Uber open-sourced Jaeger, a distributed tracing system for microservices, inspired by Google Dapper.
Uber	2017-05-02	Uber open-sourced RIBs, the cross-platform architecture framework behind many of their mobile applications.
Uber	2017-11-02	Uber open-sourced Pyro, a deep probabilistic programming language built on PyTorch.
Uber	2018-02-06	Uber open-sourced Horovod, a framework for distributed deep learning.

Apple	2018-03-14	Apple open-sourced SwiftNIO, a cross-platform asynchronous event-driven network application framework for high-performance protocol servers and clients.
SAP	2018-03-20	SAP open-sourced the Kyma project, a Kubernetes-based runtime to extend applications in a cloud-native world.
Cisco	2018-04-12	Cisco contributed to the Multus CNI project, a container network interface plugin for Kubernetes to support multiple network interfaces.
Apple	2018-04-19	Apple open-sourced FoundationDB, a distributed database.
Uber	2018-06-07	Uber open-sourced M3, a large-scale metrics platform for Prometheus, designed for high ingestion and query loads.
SAP	2018-06-20	SAP open-sourced Gardener, a Kubernetes management solution for hyperscale cloud providers.
Uber	2018-08-16	Uber open-sourced Manifold, a visual debugging tool for machine learning models.
Uber	2018-09-11	Uber open-sourced H3, a geospatial indexing system that enables efficient spatial queries on a hexagonal grid.
Uber	2018-09-14	Uber open-sourced Kepler.gl, a powerful open-source geospatial analysis tool for large-scale data sets.
SAP	2018-10-25	SAP released some components of SAP Vora as open-source, focusing on big data and analytics.
Uber	2018-12-12	Uber open-sourced AresDB, a real-time analytics database designed for fast aggregation and analysis.
Uber	2019-02-25	Uber open-sourced Ludwig, a toolbox that allows users to train and test deep learning models without writing code.
SAP	2019-04-04	SAP open-sourced parts of its Machine Learning Platform (MLP) for SAP HANA, providing tools for building and deploying machine learning models.
Microsoft	2019-05-02	Microsoft released open-source extensions for remote development in Visual Studio Code.
Microsoft	2019-05-06	Microsoft open-sourced Windows Terminal, a modern terminal application for command-line tools and shells.
Cisco	2019-05-21	Cisco contributed to the Network Service Mesh project under the CNCF (Cloud Native Computing Foundation) for cloud-native network services.
Microsoft	2019-06-11	Microsoft open-sourced React Native for Windows, enabling developers to build native Windows apps using React Native.
Cisco	2019-06-25	Cisco announced OpenRoaming, a project to create a seamless and secure public Wi-Fi experience, contributing the standard to the Wireless Broadband Alliance.
SAP	2019-07-10	SAP open-sourced UI5 Web Components to build enterprise-ready web applications using lightweight UI components.
Facebook	2019-07-11	Facebook released Hermes, a JavaScript engine optimized for React Native, as an open-source project.
Microsoft	2019-12-06	Microsoft announced that the Azure SDKs were being open-sourced, providing libraries for multiple programming languages.
Microsoft	2020-01-15	Microsoft released the Chromium-based version of Microsoft Edge as part of its move to open-source development practices.
SAP	2020-02-20	SAP open-sourced Fosstars, a framework for calculating risk scores for open-source components.
Apple	2020-06-22	Apple open-sourced the HomeKit ADK to encourage third-party development.

Apple	2020-07-10	Apple released the Password Manager Resources project to help developers create strong passwords.
Cisco	2020-10-08	Cisco integrated and enhanced ThousandEyes' capabilities, releasing tools to help monitor internet performance as open-source.
Apple	2020-11-19	Apple collaborated with Google to enhance TensorFlow's performance on macOS and open-sourced the efforts.
