# Supplementary material for: Winners and losers in the platform revolution
Source: PLoS One. 2026 Feb 10;21(2):e0340459. doi: 10.1371/journal.pone.0340459 (PMC12890110; doi:10.1371/journal.pone.0340459)
Supplement: Appendix B — (PDF) [file pone.0340459.s002.pdf]

1 Appendix B: Value-Creating Events

Table 1: Value-Creating Open-source Platform Events

Company	Date	Event	Patell-z	p-val
Apple	2015-12-03	Apple open-sourced the Swift programming language.	0.0351	0.0187
Apple	2018-03-14	Apple open-sourced SwiftNIO, a cross-platform asynchronous event-driven network application framework for high-performance protocol servers and clients.	0.5316	0.0984
Apple	2018-04-19	Apple open-sourced FoundationDB, a distributed database.	1.0428	0.0523
Google	2007-11-05	Google announced the open-sourcing of the Android OS.	0.4542	0.0909
Google	2008-09-02	Google released Chromium, the open-source project behind the Chrome browser.	0.7176	0.0460
Google	2015-03-19	Google open-sourced Bazel, a build and test tool.	1.9967	0.0220
Microsoft	2016-04-14	Microsoft open-sourced Visual Studio Code, a source code editor.	0.1595	0.0239
Microsoft	2020-01-15	Microsoft released the Chromium-based version of Microsoft Edge as part of its move to open-source development practices.	1.3700	0.0006
Microsoft	2019-12-06	Microsoft announced that the Azure SDKs were being open-sourced, providing libraries for multiple programming languages.	0.6027	0.0054
SAP	2018-10-25	SAP released some components of SAP Vora as open-source, focusing on big data and analytics.	0.6241	0.0398
Twitter	2011-08-19	Twitter open-sourced Bootstrap, a popular front-end framework for web development.	0.4241	0.0044
Twitter	2016-06-15	Twitter open-sourced Heron, a real-time analytics platform that is the successor to Apache Storm.	0.6020	0.0955
Uber	2017-04-20	Uber open-sourced Jaeger, a distributed tracing system for microservices, inspired by Google Dapper.	0.6326	0.0000
Uber	2017-05-02	Uber open-sourced RIBs, the cross-platform architecture framework behind many of their mobile applications.	0.6414	0.0000
