# Supplementary material for: Winners and losers in the platform revolution
Source: PLoS One. 2026 Feb 10;21(2):e0340459. doi: 10.1371/journal.pone.0340459 (PMC12890110; doi:10.1371/journal.pone.0340459)
Supplement: Appendix C — (PDF) [file pone.0340459.s003.pdf]

1 Appendix C: Value-Destroying Events

Table 1: Value-Destroying Open-source Platform Events

Company	Date	Event	Patell-z	p-val
Apple	2020-11-19	Apple collaborated with Google to enhance TensorFlow's performance on macOS and open-sourced the efforts.	-1.0080	0.0497
Apple	2020-06-22	Apple open-sourced the HomeKit ADK to encourage third-party development.	-2.9077	0.0000
Apple	2020-07-10	Apple released the Password Manager Resources project to help developers create strong passwords.	-3.1854	0.0000
Microsoft	2014-11-12	Microsoft open-sourced .NET Core, a cross-platform framework for building applications.	-0.0753	0.0398
Microsoft	2012-10-01	Microsoft open-sourced TypeScript, a typed superset of JavaScript.	-1.4113	0.0165
Microsoft	2016-08-18	Microsoft open-sourced PowerShell, a cross-platform task automation framework.	-0.3332	0.0923
SAP	2018-06-20	SAP open-sourced Gardener, a Kubernetes management solution for hyperscale cloud providers.	-0.4410	0.0286
SAP	2020-02-20	SAP open-sourced Fosstars, a framework for calculating risk scores for open-source components.	-1.3195	0.0000
Twitter	2011-07-18	Twitter open-sourced Finagle, an extensible RPC system for the JVM used for building asynchronous, distributed systems.	-0.2404	0.0001
Twitter	2012-08-21	Twitter open-sourced Bower, a package manager for the web.	-2.5808	0.0007
Twitter	2012-07-12	Twitter made significant contributions to the Hadoop ecosystem, particularly around scalability and integration.	-0.7821	0.0472
Twitter	2012-04-19	Twitter open-sourced Scalding, a Scala API for Cascading, which is a framework for building robust data processing workflows on Hadoop.	-0.1734	0.0359
Twitter	2012-08-14	Twitter open-sourced Zipkin, a distributed tracing system for service-oriented architectures.	-2.3197	0.0020
Twitter	2013-06-15	Twitter open-sourced Twemproxy, a fast and lightweight proxy for Memcached and Redis protocols.	-0.5203	0.0314
Uber	2017-11-02	Uber open-sourced Pyro, a deep probabilistic programming language built on PyTorch.	-0.7202	0.0041
