# Supplementary material for: Winners and losers in the platform revolution
Source: PLoS One. 2026 Feb 10;21(2):e0340459. doi: 10.1371/journal.pone.0340459 (PMC12890110; doi:10.1371/journal.pone.0340459)
Supplement: Appendix D — (PDF) [file pone.0340459.s004.pdf]

1 Appendix D: Events That Did Not Impact Firm Value

Table 1: Indetermenant Open-source Platform Events

Company	Date	Event	Patell-z	p-val
Apple	2003-06-13	Apple open-sourced WebKit, the browser engine used in Safari.	0.0163	0.1228
Apple	2015-03-09	Apple announced and released ResearchKit as an open-source framework for medical research.	0.5909	0.1955
Apple	2016-04-21	Apple released CareKit, an open-source framework for healthcare apps.	0.4391	0.1554
Apple	1999-01-01	Apple first released the source code for Darwin, the Unix-based core of macOS.	0.4916	0.1439
Google	2015-11-09	Google open-sourced TensorFlow, its machine learning framework.	1.0870	0.1290
Google	2014-06-07	Google open-sourced Kubernetes, a container orchestration platform.	-1.1543	0.1706
Google	2012-03-10	Google open-sourced the Go (Golang) programming language.	-0.5676	0.2241
Google	2010-10-20	Google open-sourced AngularJS, a web application framework.	-0.8037	0.1787
Google	2016-08-15	Google released the source code for its experimental operating system, Fuchsia.	-0.5014	0.1527
Google	2017-01-10	Google contributed the Dataflow SDK to the Apache Software Foundation, forming Apache Beam.	-1.0734	0.1963
Google	2008-07-07	Google released Protocol Buffers as an open-source project.	-0.5976	0.2487
Microsoft	2016-01-13	Microsoft open-sourced ChakraCore, the core of the Chakra JavaScript engine.	-0.1467	0.1458
Microsoft	2019-05-02	Microsoft released open-source extensions for remote development in Visual Studio Code.	-0.0689	0.2000
Microsoft	2019-06-11	Microsoft open-sourced React Native for Windows, enabling developers to build native Windows apps using React Native.	-0.7211	0.3016
Microsoft	2019-05-06	Microsoft open-sourced Windows Terminal, a modern terminal application for command-line tools and shells.	-0.1496	0.3630
SAP	2018-03-20	SAP open-sourced the Kyma project, a Kubernetes-based runtime to extend applications in a cloud-native world.	-0.4042	0.1055
SAP	2017-02-21	SAP open-sourced Project ”Piper,” a continuous integration and delivery (CI/CD) tool for DevOps.	0.4429	0.4963
SAP	2019-07-10	SAP open-sourced UI5 Web Components to build enterprise-ready web applications using lightweight UI components.	0.6230	0.3858
SAP	2016-03-24	SAP contributed the Dirigible project to the Eclipse Foundation, an open-source application development platform.	-0.0864	0.4269

SAP	2019-04-04	SAP open-sourced parts of its Machine Learning Platform (MLP) for SAP HANA, providing tools for building and deploying machine learning models.	0.5052	0.2938
Twitter	2010-06-01	Twitter began contributing to Apache Mesos, a cluster manager that provides efficient resource isolation and sharing.	0.4649	0.2582
Twitter	2016-04-27	Twitter open-sourced Cuckoo Filter, a high-performance, probabilistic data structure for fast approximate set-membership queries.	0.4014	0.2742
Uber	2018-02-06	Uber open-sourced Horovod, a framework for distributed deep learning.	0.0274	0.3016
